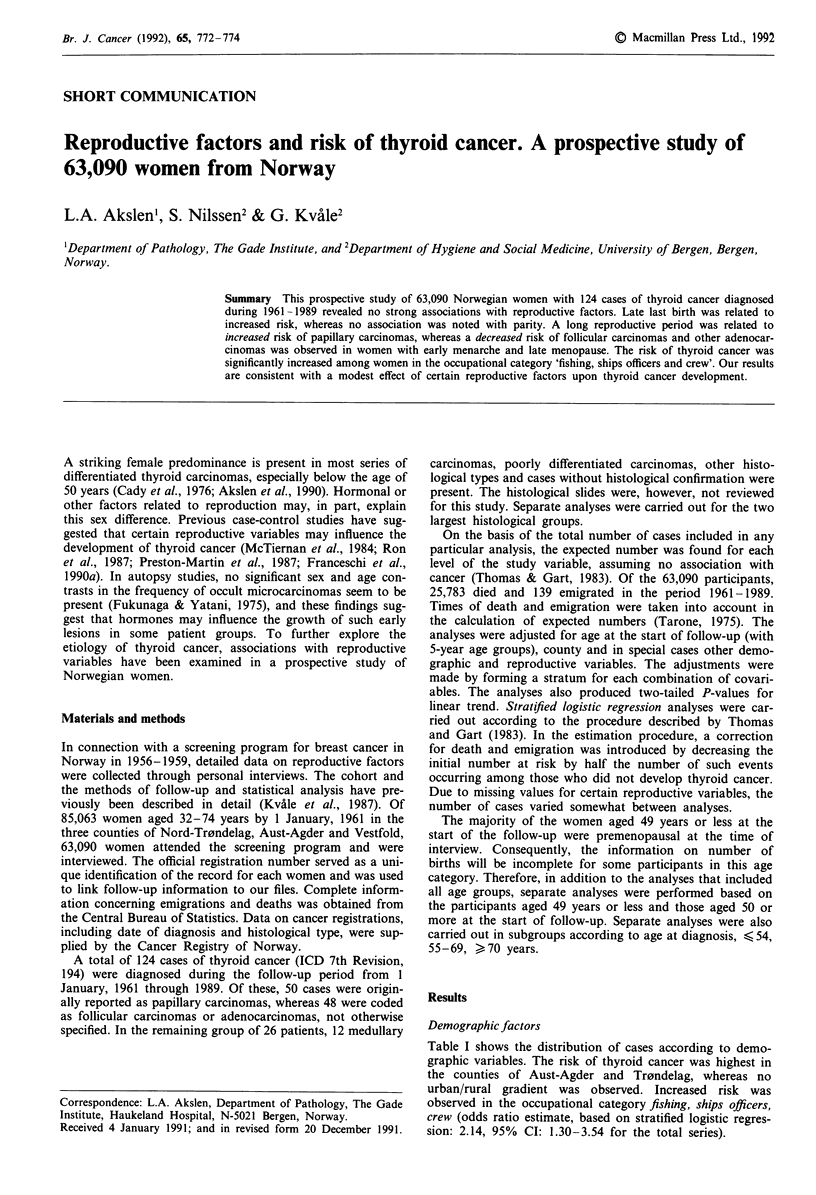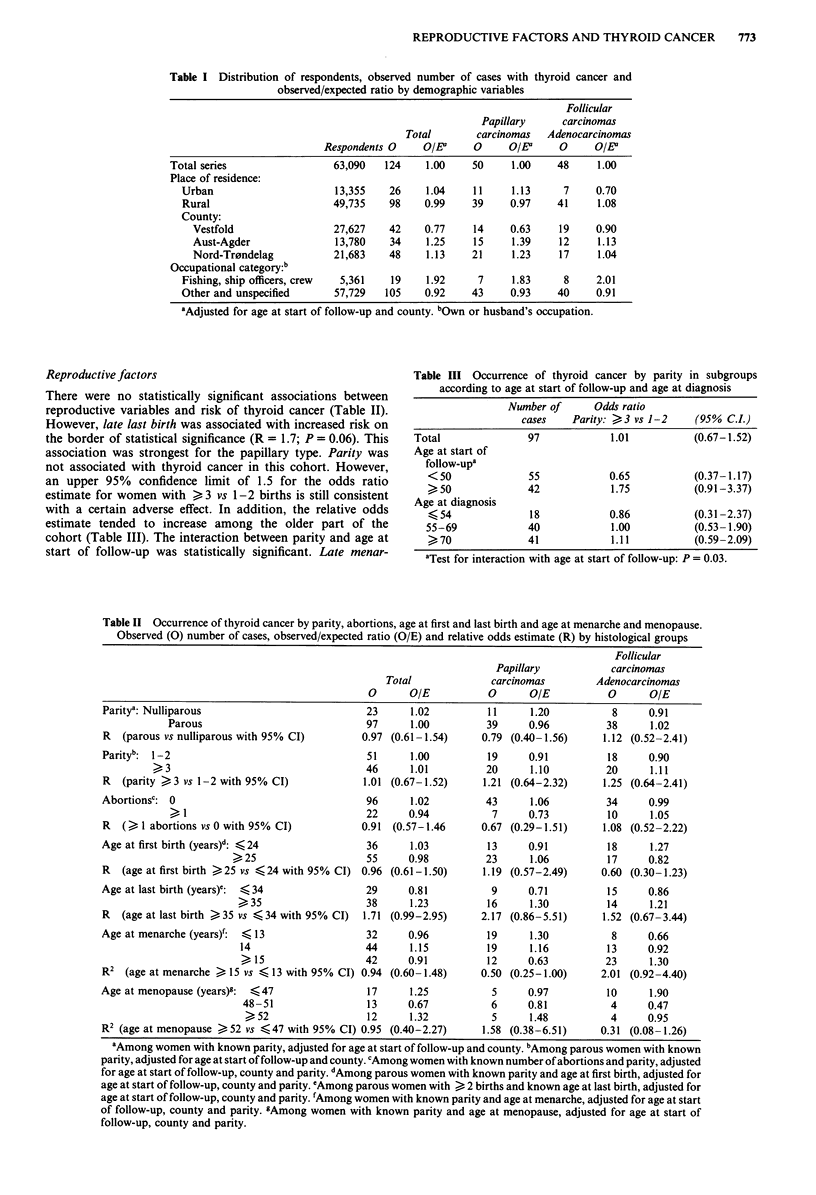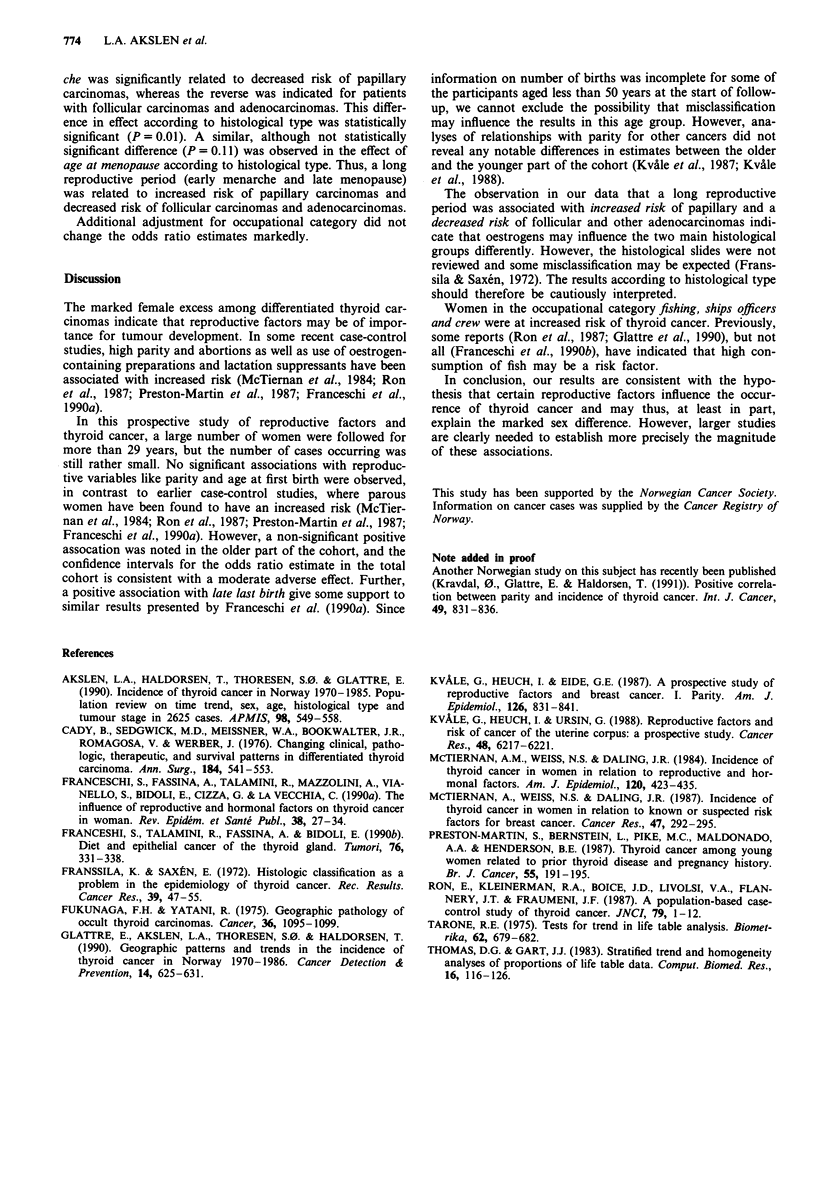# Reproductive factors and risk of thyroid cancer. A prospective study of 63,090 women from Norway.

**DOI:** 10.1038/bjc.1992.163

**Published:** 1992-05

**Authors:** L. A. Akslen, S. Nilssen, G. Kvåle

**Affiliations:** Department of Pathology, Gade Institute, Bergen, Norway.

## Abstract

This prospective study of 63,090 Norwegian women with 124 cases of thyroid cancer diagnosed during 1961-1989 revealed no strong associations with reproductive factors. Late last birth was related to increased risk, whereas no association was noted with parity. A long reproductive period was related to increased risk of papillary carcinomas, whereas a decreased risk of follicular carcinomas and other adenocarcinomas was observed in women with early menarche and late menopause. The risk of thyroid cancer was significantly increased among women in the occupational category 'fishing, ships officers and crew'. Our results are consistent with a modest effect of certain reproductive factors upon thyroid cancer development.


Br. J. Cancer (1992), 65, 772–774 © Macmillan Press Ltd., 1992

SHORT COMMUNICATION

### Reproductive factors and risk of thyroid cancer. A prospective study of 63,090 women from Norway

L.A. Akslen[1], S. Nilssen[2] & G. Kvåle[2]

[1]*Department of Pathology, The Gade Institute, and* [2]*Department of Hygiene and Social Medicine, University of Bergen, Bergen, Norway.*

**Summary** This prospective study of 63,090 Norwegian women with 124 cases of thyroid cancer diagnosed during 1961–1989 revealed no strong associations with reproductive factors. Late last birth was related to increased risk, whereas no association was noted with parity. A long reproductive period was related to *increased* risk of papillary carcinomas, whereas a *decreased* risk of follicular carcinomas and other adenocarcinomas was observed in women with early menarche and late menopause. The risk of thyroid cancer was significantly increased among women in the occupational category 'fishing, ships officers and crew'. Our results are consistent with a modest effect of certain reproductive factors upon thyroid cancer development.

A striking female predominance is present in most series of differentiated thyroid carcinomas, especially below the age of 50 years (Cady *et al.*, 1976; Akslen *et al.*, 1990). Hormonal or other factors related to reproduction may, in part, explain this sex difference. Previous case-control studies have suggested that certain reproductive variables may influence the development of thyroid cancer (McTiernan *et al.*, 1984; Ron *et al.*, 1987; Preston-Martin *et al.*, 1987; Franceschi *et al.*, 1990*a*). In autopsy studies, no significant sex and age contrasts in the frequency of occult microcarcinomas seem to be present (Fukunaga & Yatani, 1975), and these findings suggest that hormones may influence the growth of such early lesions in some patient groups. To further explore the etiology of thyroid cancer, associations with reproductive variables have been examined in a prospective study of Norwegian women.

### Materials and methods

In connection with a screening program for breast cancer in Norway in 1956–1959, detailed data on reproductive factors were collected through personal interviews. The cohort and the methods of follow-up and statistical analysis have previously been described in detail (Kvåle *et al.*, 1987). Of 85,063 women aged 32–74 years by 1 January, 1961 in the three counties of Nord-Trøndelag, Aust-Agder and Vestfold, 63,090 women attended the screening program and were interviewed. The official registration number served as a unique identification of the record for each women and was used to link follow-up information to our files. Complete information concerning emigrations and deaths was obtained from the Central Bureau of Statistics. Data on cancer registrations, including date of diagnosis and histological type, were supplied by the Cancer Registry of Norway.

A total of 124 cases of thyroid cancer (ICD 7th Revision, 194) were diagnosed during the follow-up period from 1 January, 1961 through 1989. Of these, 50 cases were originally reported as papillary carcinomas, whereas 48 were coded as follicular carcinomas or adenocarcinomas, not otherwise specified. In the remaining group of 26 patients, 12 medullary carcinomas, poorly differentiated carcinomas, other histological types and cases without histological confirmation were present. The histological slides were, however, not reviewed for this study. Separate analyses were carried out for the two largest histological groups.

On the basis of the total number of cases included in any particular analysis, the expected number was found for each level of the study variable, assuming no association with cancer (Thomas & Gart, 1983). Of the 63,090 participants, 25,783 died and 139 emigrated in the period 1961–1989. Times of death and emigration were taken into account in the calculation of expected numbers (Tarone, 1975). The analyses were adjusted for age at the start of follow-up (with 5-year age groups), county and in special cases other demographic and reproductive variables. The adjustments were made by forming a stratum for each combination of covariables. The analyses also produced two-tailed *P*-values for linear trend. *Stratified logistic regression* analyses were carried out according to the procedure described by Thomas and Gart (1983). In the estimation procedure, a correction for death and emigration was introduced by decreasing the initial number at risk by half the number of such events occurring among those who did not develop thyroid cancer. Due to missing values for certain reproductive variables, the number of cases varied somewhat between analyses.

The majority of the women aged 49 years or less at the start of the follow-up were premenopausal at the time of interview. Consequently, the information on number of births will be incomplete for some participants in this age category. Therefore, in addition to the analyses that included all age groups, separate analyses were performed based on the participants aged 49 years or less and those aged 50 or more at the start of follow-up. Separate analyses were also carried out in subgroups according to age at diagnosis, $\leqslant 54$, 55–69, $\geqslant 70$ years.

### Results

### *Demographic factors*

Table I shows the distribution of cases according to demographic variables. The risk of thyroid cancer was highest in the counties of Aust-Agder and Trøndelag, whereas no urban/rural gradient was observed. Increased risk was observed in the occupational category *fishing, ships officers, crew* (odds ratio estimate, based on stratified logistic regression: 2.14, 95% CI: 1.30–3.54 for the total series).

Correspondence: L.A. Akslen, Department of Pathology, The Gade Institute, Haukeland Hospital, N-5021 Bergen, Norway.
Received 4 January 1991; and in revised form 20 December 1991.

**Table I** Distribution of respondents, observed number of cases with thyroid cancer and observed/expected ratio by demographic variables

| | | Total | | Papillary carcinomas | | Follicular carcinomas Adenocarcinomas | |
|---|---|---|---|---|---|---|---|
| | Respondents | O | O/E[a] | O | O/E[a] | O | O/E[a] |
| Total series | 63,090 | 124 | 1.00 | 50 | 1.00 | 48 | 1.00 |
| Place of residence: | | | | | | | |
| Urban | 13,355 | 26 | 1.04 | 11 | 1.13 | 7 | 0.70 |
| Rural | 49,735 | 98 | 0.99 | 39 | 0.97 | 41 | 1.08 |
| County: | | | | | | | |
| Vestfold | 27,627 | 42 | 0.77 | 14 | 0.63 | 19 | 0.90 |
| Aust-Agder | 13,780 | 34 | 1.25 | 15 | 1.39 | 12 | 1.13 |
| Nord-Trøndelag | 21,683 | 48 | 1.13 | 21 | 1.23 | 17 | 1.04 |
| Occupational category:[b] | | | | | | | |
| Fishing, ship officers, crew | 5,361 | 19 | 1.92 | 7 | 1.83 | 8 | 2.01 |
| Other and unspecified | 57,729 | 105 | 0.92 | 43 | 0.93 | 40 | 0.91 |

[a]Adjusted for age at start of follow-up and county. [b]Own or husband's occupation.

*Reproductive factors*

There were no statistically significant associations between reproductive variables and risk of thyroid cancer (Table II). However, *late last birth* was associated with increased risk on the border of statistical significance (R = 1.7; $P = 0.06$). This association was strongest for the papillary type. *Parity* was not associated with thyroid cancer in this cohort. However, an upper 95% confidence limit of 1.5 for the odds ratio estimate for women with ⩾3 *vs* 1–2 births is still consistent with a certain adverse effect. In addition, the relative odds estimate tended to increase among the older part of the cohort (Table III). The interaction between parity and age at start of follow-up was statistically significant. *Late menar-*

**Table III** Occurrence of thyroid cancer by parity in subgroups according to age at start of follow-up and age at diagnosis

| | Number of cases | Odds ratio Parity: ⩾3 vs 1–2 | (95% C.I.) |
|---|---|---|---|
| Total | 97 | 1.01 | (0.67–1.52) |
| Age at start of follow-up[a] | | | |
| <50 | 55 | 0.65 | (0.37–1.17) |
| ⩾50 | 42 | 1.75 | (0.91–3.37) |
| Age at diagnosis | | | |
| ⩽54 | 18 | 0.86 | (0.31–2.37) |
| 55–69 | 40 | 1.00 | (0.53–1.90) |
| ⩾70 | 41 | 1.11 | (0.59–2.09) |

[a]Test for interaction with age at start of follow-up: $P = 0.03$.

**Table II** Occurrence of thyroid cancer by parity, abortions, age at first and last birth and age at menarche and menopause. Observed (O) number of cases, observed/expected ratio (O/E) and relative odds estimate (R) by histological groups

| | Total | | Papillary carcinomas | | Follicular carcinomas Adenocarcinomas | |
|---|---|---|---|---|---|---|
| | O | O/E | O | O/E | O | O/E |
| Parity[a]: Nulliparous | 23 | 1.02 | 11 | 1.20 | 8 | 0.91 |
| Parous | 97 | 1.00 | 39 | 0.96 | 38 | 1.02 |
| R (parous *vs* nulliparous with 95% CI) | 0.97 | (0.61–1.54) | 0.79 | (0.40–1.56) | 1.12 | (0.52–2.41) |
| Parity[b]: 1–2 | 51 | 1.00 | 19 | 0.91 | 18 | 0.90 |
| ⩾3 | 46 | 1.01 | 20 | 1.10 | 20 | 1.11 |
| R (parity ⩾3 *vs* 1–2 with 95% CI) | 1.01 | (0.67–1.52) | 1.21 | (0.64–2.32) | 1.25 | (0.64–2.41) |
| Abortions[c]: 0 | 96 | 1.02 | 43 | 1.06 | 34 | 0.99 |
| ⩾1 | 22 | 0.94 | 7 | 0.73 | 10 | 1.05 |
| R (⩾1 abortions *vs* 0 with 95% CI) | 0.91 | (0.57–1.46 | 0.67 | (0.29–1.51) | 1.08 | (0.52–2.22) |
| Age at first birth (years)[d]: ⩽24 | 36 | 1.03 | 13 | 0.91 | 18 | 1.27 |
| ⩾25 | 55 | 0.98 | 23 | 1.06 | 17 | 0.82 |
| R (age at first birth ⩾25 *vs* ⩽24 with 95% CI) | 0.96 | (0.61–1.50) | 1.19 | (0.57–2.49) | 0.60 | (0.30–1.23) |
| Age at last birth (years)[e]: ⩽34 | 29 | 0.81 | 9 | 0.71 | 15 | 0.86 |
| ⩾35 | 38 | 1.23 | 16 | 1.30 | 14 | 1.21 |
| R (age at last birth ⩾35 *vs* ⩽34 with 95% CI) | 1.71 | (0.99–2.95) | 2.17 | (0.86–5.51) | 1.52 | (0.67–3.44) |
| Age at menarche (years)[f]: ⩽13 | 32 | 0.96 | 19 | 1.30 | 8 | 0.66 |
| 14 | 44 | 1.15 | 19 | 1.16 | 13 | 0.92 |
| ⩾15 | 42 | 0.91 | 12 | 0.63 | 23 | 1.30 |
| $R^2$ (age at menarche ⩾15 *vs* ⩽13 with 95% CI) | 0.94 | (0.60–1.48) | 0.50 | (0.25–1.00) | 2.01 | (0.92–4.40) |
| Age at menopause (years)[g]: ⩽47 | 17 | 1.25 | 5 | 0.97 | 10 | 1.90 |
| 48–51 | 13 | 0.67 | 6 | 0.81 | 4 | 0.47 |
| ⩾52 | 12 | 1.32 | 5 | 1.48 | 4 | 0.95 |
| $R^2$ (age at menopause ⩾52 *vs* ⩽47 with 95% CI) | 0.95 | (0.40–2.27) | 1.58 | (0.38–6.51) | 0.31 | (0.08–1.26) |

[a]Among women with known parity, adjusted for age at start of follow-up and county. [b]Among parous women with known parity, adjusted for age at start of follow-up and county. [c]Among women with known number of abortions and parity, adjusted for age at start of follow-up, county and parity. [d]Among parous women with known parity and age at first birth, adjusted for age at start of follow-up, county and parity. [e]Among parous women with ⩾2 births and known age at last birth, adjusted for age at start of follow-up, county and parity. [f]Among women with known parity and age at menarche, adjusted for age at start of follow-up, county and parity. [g]Among women with known parity and age at menopause, adjusted for age at start of follow-up, county and parity.

*che* was significantly related to decreased risk of papillary carcinomas, whereas the reverse was indicated for patients with follicular carcinomas and adenocarcinomas. This difference in effect according to histological type was statistically significant ($P = 0.01$). A similar, although not statistically significant difference ($P = 0.11$) was observed in the effect of *age at menopause* according to histological type. Thus, a long reproductive period (early menarche and late menopause) was related to increased risk of papillary carcinomas and decreased risk of follicular carcinomas and adenocarcinomas.

Additional adjustment for occupational category did not change the odds ratio estimates markedly.

### Discussion

The marked female excess among differentiated thyroid carcinomas indicate that reproductive factors may be of importance for tumour development. In some recent case-control studies, high parity and abortions as well as use of oestrogen-containing preparations and lactation suppressants have been associated with increased risk (McTiernan *et al.*, 1984; Ron *et al.*, 1987; Preston-Martin *et al.*, 1987; Franceschi *et al.*, 1990*a*).

In this prospective study of reproductive factors and thyroid cancer, a large number of women were followed for more than 29 years, but the number of cases occurring was still rather small. No significant associations with reproductive variables like parity and age at first birth were observed, in contrast to earlier case-control studies, where parous women have been found to have an increased risk (McTiernan *et al.*, 1984; Ron *et al.*, 1987; Preston-Martin *et al.*, 1987; Franceschi *et al.*, 1990*a*). However, a non-significant positive assocation was noted in the older part of the cohort, and the confidence intervals for the odds ratio estimate in the total cohort is consistent with a moderate adverse effect. Further, a positive association with *late last birth* give some support to similar results presented by Franceschi *et al.* (1990*a*). Since information on number of births was incomplete for some of the participants aged less than 50 years at the start of follow-up, we cannot exclude the possibility that misclassification may influence the results in this age group. However, analyses of relationships with parity for other cancers did not reveal any notable differences in estimates between the older and the younger part of the cohort (Kvåle *et al.*, 1987; Kvåle *et al.*, 1988).

The observation in our data that a long reproductive period was associated with *increased risk* of papillary and a *decreased risk* of follicular and other adenocarcinomas indicate that oestrogens may influence the two main histological groups differently. However, the histological slides were not reviewed and some misclassification may be expected (Franssila & Saxén, 1972). The results according to histological type should therefore be cautiously interpreted.

Women in the occupational category *fishing, ships officers and crew* were at increased risk of thyroid cancer. Previously, some reports (Ron *et al.*, 1987; Glattre *et al.*, 1990), but not all (Franceschi *et al.*, 1990*b*), have indicated that high consumption of fish may be a risk factor.

In conclusion, our results are consistent with the hypothesis that certain reproductive factors influence the occurrence of thyroid cancer and may thus, at least in part, explain the marked sex difference. However, larger studies are clearly needed to establish more precisely the magnitude of these associations.

This study has been supported by the *Norwegian Cancer Society*. Information on cancer cases was supplied by the *Cancer Registry of Norway*.

**Note added in proof**

Another Norwegian study on this subject has recently been published (Kravdal, Ø., Glattre, E. & Haldorsen, T. (1991)). Positive correlation between parity and incidence of thyroid cancer. *Int. J. Cancer*, **49**, 831–836.

### References

AKSLEN, L.A., HALDORSEN, T., THORESEN, S.Ø. & GLATTRE, E. (1990). Incidence of thyroid cancer in Norway 1970–1985. Population review on time trend, sex, age, histological type and tumour stage in 2625 cases. *APMIS*, **98**, 549–558.

CADY, B., SEDGWICK, M.D., MEISSNER, W.A., BOOKWALTER, J.R., ROMAGOSA, V. & WERBER, J. (1976). Changing clinical, pathologic, therapeutic, and survival patterns in differentiated thyroid carcinoma. *Ann. Surg.*, **184**, 541–553.

FRANCESCHI, S., FASSINA, A., TALAMINI, R., MAZZOLINI, A., VIANELLO, S., BIDOLI, E., CIZZA, G. & LA VECCHIA, C. (1990*a*). The influence of reproductive and hormonal factors on thyroid cancer in woman. *Rev. Epidém. et Santé Publ.*, **38**, 27–34.

FRANCESHI, S., TALAMINI, R., FASSINA, A. & BIDOLI, E. (1990*b*). Diet and epithelial cancer of the thyroid gland. *Tumori*, **76**, 331–338.

FRANSSILA, K. & SAXÉN, E. (1972). Histologic classification as a problem in the epidemiology of thyroid cancer. *Rec. Results. Cancer Res.*, **39**, 47–55.

FUKUNAGA, F.H. & YATANI, R. (1975). Geographic pathology of occult thyroid carcinomas. *Cancer*, **36**, 1095–1099.

GLATTRE, E., AKSLEN, L.A., THORESEN, S.Ø. & HALDORSEN, T. (1990). Geographic patterns and trends in the incidence of thyroid cancer in Norway 1970–1986. *Cancer Detection & Prevention*, **14**, 625–631.

KVÅLE, G., HEUCH, I. & EIDE, G.E. (1987). A prospective study of reproductive factors and breast cancer. I. Parity. *Am. J. Epidemiol.*, **126**, 831–841.

KVÅLE, G., HEUCH, I. & URSIN, G. (1988). Reproductive factors and risk of cancer of the uterine corpus: a prospective study. *Cancer Res.*, **48**, 6217–6221.

MCTIERNAN, A.M., WEISS, N.S. & DALING, J.R. (1984). Incidence of thyroid cancer in women in relation to reproductive and hormonal factors. *Am. J. Epidemiol.*, **120**, 423–435.

MCTIERNAN, A., WEISS, N.S. & DALING, J.R. (1987). Incidence of thyroid cancer in women in relation to known or suspected risk factors for breast cancer. *Cancer Res.*, **47**, 292–295.

PRESTON-MARTIN, S., BERNSTEIN, L., PIKE, M.C., MALDONADO, A.A. & HENDERSON, B.E. (1987). Thyroid cancer among young women related to prior thyroid disease and pregnancy history. *Br. J. Cancer*, **55**, 191–195.

RON, E., KLEINERMAN, R.A., BOICE, J.D., LIVOLSI, V.A., FLANNERY, J.T. & FRAUMENI, J.F. (1987). A population-based case-control study of thyroid cancer. *JNCI*, **79**, 1–12.

TARONE, R.E. (1975). Tests for trend in life table analysis. *Biometrika*, **62**, 679–682.

THOMAS, D.G. & GART, J.J. (1983). Stratified trend and homogeneity analyses of proportions of life table data. *Comput. Biomed. Res.*, **16**, 116–126.